# Oxidative damage induced by cigarette smoke exposure in mice: impact on lung tissue and diaphragm muscle*,^**^

**DOI:** 10.1590/S1806-37132014000400009

**Published:** 2014

**Authors:** Samanta Portão de Carlos, Alexandre Simões Dias, Luiz Alberto Forgiarini, Patrícia Damiani Patricio, Thaise Graciano, Renata Tiscoski Nesi, Samuel Valença, Adriana Meira Guntzel Chiappa, Gerson Cipriano, Claudio Teodoro de Souza, Gaspar Rogério da Silva Chiappa

**Affiliations:** Department of Physical Therapy. University of Southern Santa Catarina, Criciúma, Brazil; Graduate Program in Movement Sciences and Pulmonology Sciences, Federal University of Rio Grande do Sul, Porto Alegre, Brazil; Methodist University, Instituto Porto Alegre (IPA, Porto Alegre Institute), Porto Alegre, Brazil; Department of Physical Therapy. University of Southern Santa Catarina, Criciúma, Brazil; Department of Physical Therapy. University of Southern Santa Catarina, Criciúma, Brazil; Institute of Biomedical Science, Federal University of Rio de Janeiro, Rio de Janeiro, Brazil; Laboratory for Research in the Physiopathology of Exercise, Department of Cardiology, Porto Alegre Hospital de Clínicas, Porto Alegre, Brazil; Intensive Care Unit, Porto Alegre Hospital de Clínicas, Porto Alegre, Brazil; Health Sciences and Technologies Program, Department of Physical Therapy, University of Brasília, Brasília, Brazil; Department of Physical Therapy. University of Southern Santa Catarina, Criciúma, Brazil; Laboratory for Research in the Physiopathology of Exercise, Department of Cardiology, Porto Alegre Hospital de Clínicas, Porto Alegre, Brazil; and Epidemiology and Public Health Research Group, Serra Gaucha College, Caxias do Sul, Brazil

**Keywords:** Oxidative stress, Mice, Respiratory system, Smoking, Inflammation

## Abstract

**OBJECTIVE:**

To evaluate oxidative damage (lipid oxidation, protein oxidation, thiobarbituric acid-reactive substances [TBARS], and carbonylation) and inflammation (expression of phosphorylated AMP-activated protein kinase and mammalian target of rapamycin [p-AMPK and p-mTOR, respectively]) in the lung parenchyma and diaphragm muscles of male C57BL-6 mice exposed to cigarette smoke (CS) for 7, 15, 30, 45, or 60 days.

**METHODS:**

Thirty-six male C57BL-6 mice were divided into six groups (n = 6/group): a control group; and five groups exposed to CS for 7, 15, 30, 45, and 60 days, respectively.

**RESULTS:**

Compared with control mice, CS-exposed mice presented lower body weights at 30 days. In CS-exposed mice (compared with control mice), the greatest differences (increases) in TBARS levels were observed on day 7 in diaphragm-muscle, compared with day 45 in lung tissue; the greatest differences (increases) in carbonyl levels were observed on day 7 in both tissue types; and sulfhydryl levels were lower, in both tissue types, at all time points. In lung tissue and diaphragm muscle, p-AMPK expression exhibited behavior similar to that of TBARS. Expression of p-mTOR was higher than the control value on days 7 and 15 in lung tissue, as it was on day 45 in diaphragm muscle.

**CONCLUSION:**

Our data demonstrate that CS exposure produces oxidative damage, not only in lung tissue but also (primarily) in muscle tissue, having an additional effect on respiratory muscle, as is frequently observed in smokers with COPD.

## Introduction

Cigarette smoke (CS) contains a large number of oxidants that have adverse effects on tissues through oxidative damage.^(^ 1 ^,^ 2 ^)^ It is known that CS activates inflammatory cells, which can also increase polymorphonuclear cell production of oxidants in tissues, triggering oxidative stress, a crucial step in the pathogenesis of CS-induced tissue damage.^(^ 3 ^-^ 6 ^)^ The combined effects of greater proteolytic damage, increased cell death, and decreased lung remodeling leads to emphysematous changes in the lungs.^(^ 7 ^)^ Studies have shown that, in the blood of smokers,^(^ 8 ^,^ 9 ^)^ as well as in various organs of animals chronically exposed to CS,^(^ 10 ^)^ there are increases in lipid peroxidation, protein carbonylation, thiol oxidation, and DNA oxidization. 

There is evidence that two central factors are involved in CS-induced direct injury or systemic inflammation: phosphorylated AMP-activated protein kinase and phosphorylated mammalian target of rapamycin (p-AMPK and p-mTOR, respectively). One recent study showed that p-AMPK activation inhibits or promotes inflammation, depending on the stimulus.^(^ 11 ^)^ There is also increasing evidence that, in many cell types, an increase in intracellular reactive oxygen species (ROS) can activate p-AMPK. ^(^ 12 ^)^ A major integrator of environmental cues, mTOR controls cellular metabolism, growth, proliferation, and survival depending on mitogenic signals, as well as on the availability of nutrients and energy. It has now become clear that mTOR signaling plays a central role in regulating basic aspects of cell and organism behavior, and its dysregulation is strongly associated with progression of numerous human proliferative and metabolic diseases, including cancer, obesity, type 2 diabetes, and hamartoma syndrome.^(^ 13 ^)^

It is of great importance to elucidate the possible oxidative damage induced by CS directly in skeletal muscle, as well as the related structural abnormalities and the direct relationship between p-AMPK and p-mTOR, two factors associated with inflammation. Therefore, the aim of this animal study was to evaluate oxidative damage and inflammation in the lung parenchyma and diaphragm after 7, 15, 30, 45, and 60 days of exposure to CS.

## Methods

In this study, we used 36 two-month-old male C57BL/6 mice weighing 30-35 g. The animals were used and cared for in accordance with European Communities Council Directive 86/609/EEC of 24 November, 1986. The procedures adopted in this study were approved by the Research Ethics Committee of the University of Southern Santa Catarina, in the city of Criciúma, Brazil. The mice were housed in a temperature- and humidity-controlled environment (70% humidity; 20 ± 2°C), on a 12/12-h light/dark cycle, and were given *ad libitum* access to water and chow (Nuvilab CR1; Nuvital Nutrientes Ltda., Colombo, Brazil). The animals were checked periodically in order to verify that they remained pathogen-free. For biochemical assays, the mice were randomized into six groups (n = 6/group): a control group; and five groups exposed to CS for 7, 15, 30, 45, and 60 days (designated CS-7, CS-15, CS-30, CS-45, and CS-60, respectively).

We used commercial filter cigarettes (Marlboro^TM^ Red, 8 mg of tar and 0.6 mg of nicotine per cigarette; Philip Morris Products, Richmond, VA, USA).^(^ 14 ^,^ 15 ^)^ Study animals were exposed to the smoke emitted from the burning of 12 cigarettes per day for 7, 15, 30, 45, and 60 days, as described previously by Menegali et al.^(^ 3 ^)^ In brief, animals were placed in a covered inhalation chamber (40 cm long, 30 cm wide, and 25 cm high), positioned under an exhaust hood. A cigarette was coupled to a plastic 60-mL syringe so that each puff could be drawn in and subsequently expelled into the exposure chamber. One liter of smoke (20 puffs of 50 mL) was aspirated from each cigarette, each puff being immediately injected into the inhalation chamber. The animals were maintained in this smoke-air condition (3% smoke) for 6 min. We then removed the cover from the inhalation chamber and turned on the exhaust hood, which evacuated the smoke within 60 s. This process was immediately repeated. A total of four cigarettes were thus "smoked" in each treatment. The mice were subjected to these four-cigarette treatments three times per day (morning, noon, and afternoon), resulting in 72 min of CS exposure (12 cigarettes per day). ^(^ 16 ^)^ Each cigarette smoked produced 300 mg/m^3^ of total particulate matter in the exposure chamber.^(^ 3 ^)^ The animals were sacrificed by cervical dislocation at 24 h after the final CS exposure. Samples of lung tissue and diaphragm muscle were homogenized in buffer solution. The homogenates were centrifuged at 1000 × *g* for 10 min at 4°C, and the supernatants were stored at −70°C for subsequent use in the experiments.

For histological analysis, were selected all animals in each group. The right ventricle was perfused with sterile saline (0.9%) to remove blood from the lung. The right lung was fixed (by gentle infusion of 4% phosphate buffered formalin (pH 7.2) at 25 cmH_2_O for 2 min through a tracheal catheter), after which it was removed and weighed. Inflated lungs were fixed for 48 h and then embedded in paraffin. Serial sagittal sections (5-µm) were obtained for histological and morphometric analyses. Macrophages and neutrophils were quantified in the alveoli. For each group, were analyzed 30 microscopic fields (10 random fields, of 26,000 mm^2^ each, in 3 different sections of the right lung). The number of macrophages and neutrophils (cells/mm^2)^ were counted in a fluorescence microscope (BH-2; Olympus, Tokyo, Japan) equipped with a 40× objective.^(^ 3 ^)^

Oxidative damage was evaluated by quantifying sulfhydryl content, protein carbonyls, and malondialdehyde. Total thiol content was determined using the 5,50-dithiobis (2-nitrobenzoic acid)-DTNB-method (Sigma, St. Louis, MO, USA). The conditions of the DTNB test were as previously described.^(^ 17 ^)^ In brief, 30 µL of a sample was mixed with 1 mL of PBS and 1 mM of EDTA (pH 7.5). The reaction was started by the addition of 30 µL of 10 mM DTNB stock solution in PBS. Control samples, which did not include DTNB or protein, were run simultaneously. After 30 min of incubation at room temperature, the absorbance was read at 412 nm and the amounts of 5-thio-2-nitrobenzoic acid (TNB) formed (equivalent to the amount of sulfhydryl groups) were measured. Protein carbonyls were determined using the 2,4-dinitrophenylhydrazine (DNPH) spectrophotometry method, as described by Levine et al.^(^ 18 ^)^ In brief, samples containing either 2 N hydrochloric acid or DNPH were passed through columns containing Sephadex G-10 and rinsed with 2 N hydrochloric acid. The effluent was collected and mixed with guanidine hydrochloride, after which the absorbance determined at 360 nm in a spectrophotometer (SP 1105; Shanghai Spectrum Instruments Co., Ltd., Shanghai, China). The difference in absorbance with and without DNPH was calculated for all samples. Values are expressed as molar quantities using the extinction coefficient 22,000 [M-1]. Malondialdehyde, an important indicator of lipid peroxidation, was determined by spectrophotometry of the pink-colored product of thiobarbituric acid-reactive substances (TBARS). Total TBARS, as a proxy for lipid peroxidation (malondialdehyde levels), are expressed as mmol/mg of protein.^(^ 19 ^)^

Western blotting, the lung homogenates were prepared from the frozen lungs using a tissue lysis buffer (50 mM TRIS, pH 8.0, 5 mM EDTA, 150 mM NaCl, 1% nonionic detergent, 0.5% sodium deoxycholate, and 0.1% sodium dodecyl sulfate) and a protease inhibitor cocktail (Sigma). The lysates were clarified by centrifugation at 13,000 g for 15 min at 4°C; 10-30 g of protein were separated by SDS-PAGE on 10% or 15% gels; and p-AMPK and p-mTOR expression (antibodies from Cell Signaling Biotechnology, Boston, MA, USA) was analyzed by immunoblot analysis. Immunoreactivity was detected by enhanced chemiluminescence (ECL; Amersham Biosciences, Buckinghamshire, UK). The band density was determined using an imaging densitometer and analyzed with the accompanying software (GS-700 and Quantity One; Bio-Rad Laboratories, Hercules, CA, USA).^(^ 20 ^)^

Data are expressed as mean ± standard error of the mean. To compare means between and among groups, we used one-way ANOVA followed by Tukey's honestly significant difference post-hoc test for multiple comparisons. The level of significance was set at p < 0.05. The software used for analysis of the data was the Statistical Package for the Social Sciences, version 18.0 for Windows (SPSS Inc., Chicago, IL, USA). The sample size was based on previous studies performed in our laboratory,^(^ 3 ^)^ in which similar approaches were employed.

## Results

Among the mice evaluated in the present study, the survival rate was 100%. In comparison with the baseline values, the body weights of the animals decreased after 30, 45, and 60 days of CS exposure (27 ± 1 vs. 23 ± 0.8 g; p <0.01, 26 ± 0.5 vs. 22 ± 0.4 g; p < 0.01, and 25 ± 0.7 vs. 20 ± 0.3 g; p < 0.001, respectively). In addition, the body weights of the CS-60 group mice were significantly lower than were those of the control mice, as well as being significantly lower than were those of the CS-30 and CS-45 group mice (p < 0.001 for all).

**Figure 1:**
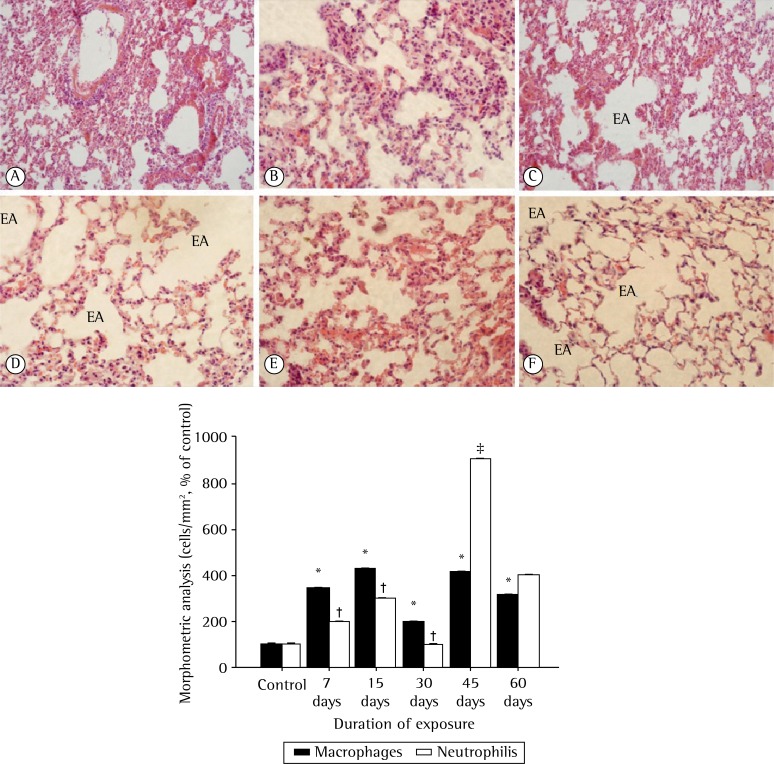
In A, photomicrographs of lung tissue samples obtained from mice exposed to cigarette smoke, showing enlarged airspaces (EAs) resulting from alveolar consolidation during the development of pulmonary emphysema (magnification, ×40): a, control group; b, 7-day exposure group; c, 15-day exposure group; d, 30-day exposure group; e, 45-day exposure group; and f, 60-day exposure group. In B, Mean ± SEM of macrophages and neutrophils (cells/mm2). *p < 0.001 vs. control for macrophages. †p < 0.001 vs. control for neutrophils. ‡p < 0.001 vs. baseline for neutrophils.

In the histological analysis, lung tissue samples obtained from control mice showed thin alveolar septa and normal alveoli, whereas those obtained from mice that were exposed to CS showed destruction of the alveolar septa (starting on day 15 of exposure), alveolar enlargement, and the presence of alveolar macrophages (Figure 1A). The alveolar enlargement was significantly greater in the CS-45 group (Figure 1A). As shown in Figure 1B, the numbers of macrophages and neutrophils in the CS groups both increased significantly (in comparison with those observed for the control group) by day 7 of exposure to CS (p < 0.01). However, the difference in the number of neutrophils was more pronounced after 45 days of exposure (p < 0.001).

**Figure 2:**
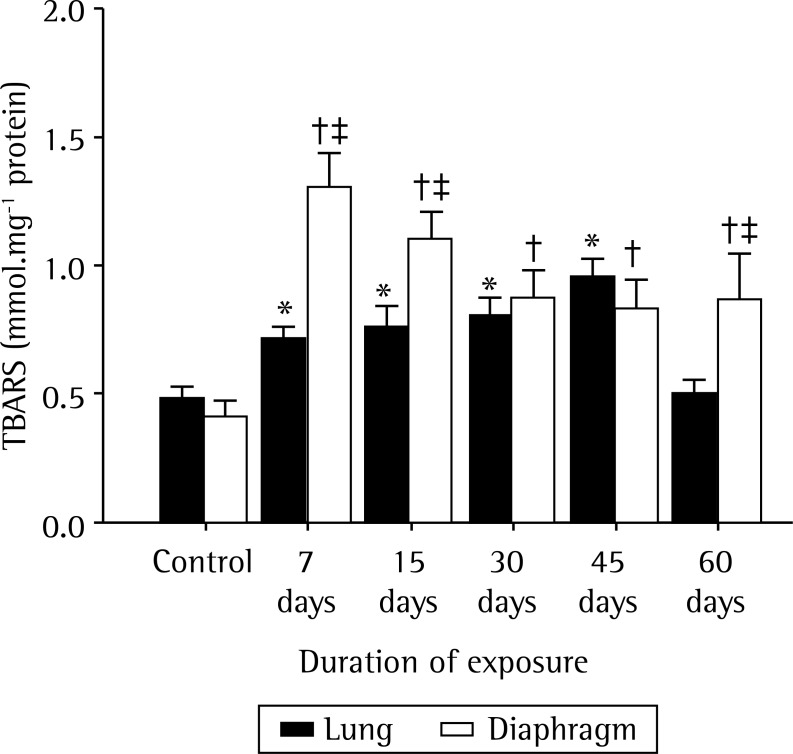
Mean ± SEM of thiobarbituric acid-reactive substances (TBARS) in lung tissue and diaphragm muscle in six groups of mice: a control group; and five groups exposed to cigarette smoke for 7, 15, 30, 45, and 60 days, respectively. *p < 0.05 vs. control in lung tissue. † p < 0.05 vs. control in diaphragm muscle. ‡ p < 0.05 vs. lung tissue.

**Figure 3:**
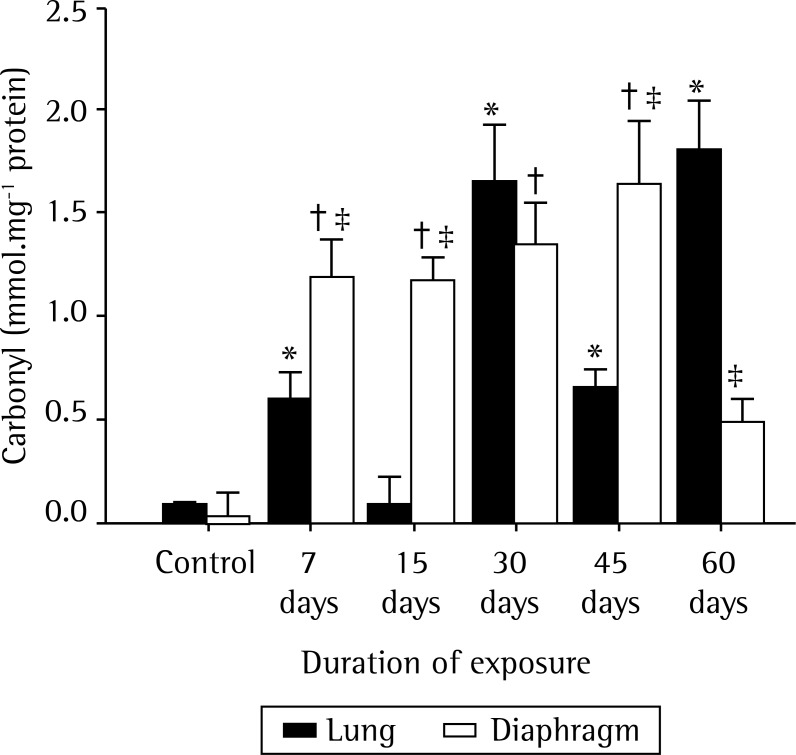
Mean ± SEM of carbonyl in lung tissue and diaphragm muscle in six groups of mice: a control group; and five groups exposed to cigarette smoke for 7, 15, 30, 45, and 60 days, respectively. * p < 0.05 vs. control in lung tissue. † p < 0.05 vs. control in diaphragm muscle. ‡ p < 0.05 vs. lung tissue.

**Figure 4:**
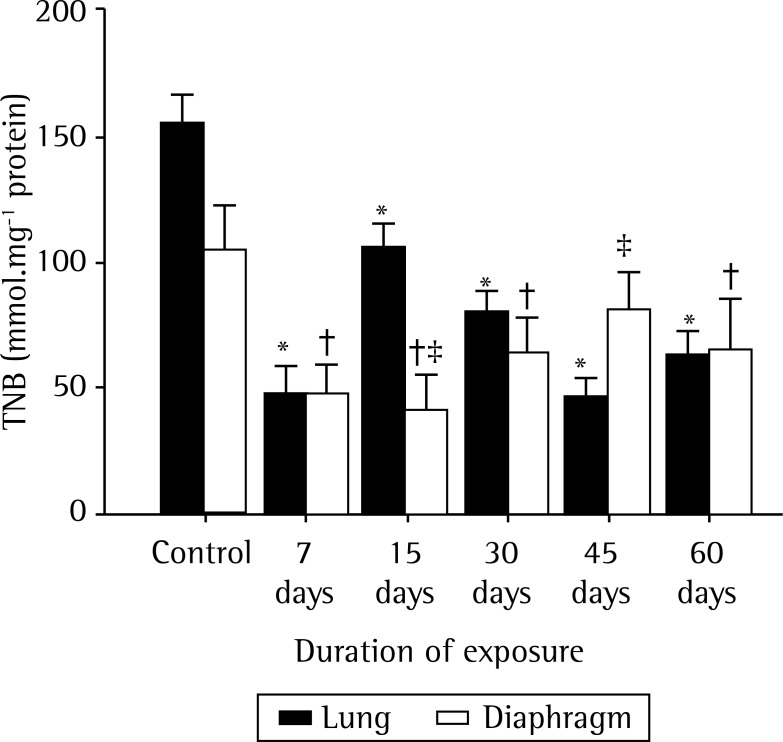
Mean ± SEM of 5-thio-2-nitrobenzoic acid (TNB) in lung tissue and diaphragm muscle in six groups of mice: a control group; and five groups exposed to cigarette smoke for 7, 15, 30, 45, and 60 days, respectively. * p < 0.05 vs. control in lung tissue. † p < 0.05 vs. control in diaphragm muscle. ‡ p < 0.05 vs. lung tissue.

Figures 2, 3, and 4, respectively, show lipid peroxidation, protein carbonyls and sulfhydryl content in lung tissue samples and diaphragm muscle samples. In both tissue types, total TBARS increased after 7 days of exposure to CS, as did carbonyl levels. In the CS-7, CS-15, and CS-45 groups, there were differences between the lung tissue samples and diaphragm muscle samples, in terms of the degree to which carbonyl levels were increased. In the CS-15 group, the levels of TNB were significantly lower in lung tissue than in diaphragm muscle. However, by day 7 of CS exposure, TNB levels were lower than the control values in both tissue types. 

**Figure 5:**
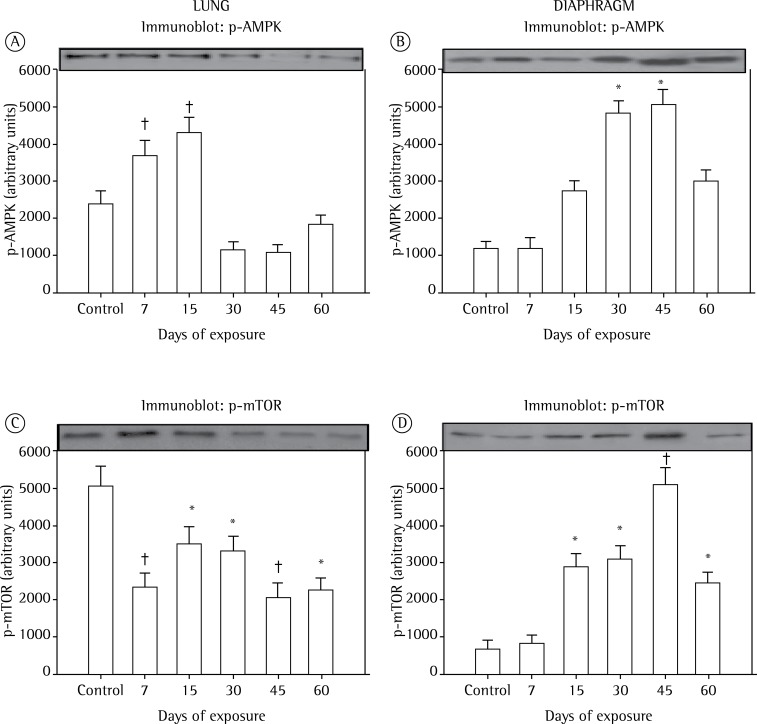
In A and B, mean ± SEM for phosphorylated AMP-activated protein kinase (p-AMPK) expression in lung tissue and diaphragm muscle, respectively. In C and D, mean ± SEM for phosphorylated mammalian target of rapamycin (p-mTOR) expression in lung tissue and diaphragm muscle, respectively. Data are related to six groups of mice: a control group; and five groups exposed to cigarette smoke for 7, 15, 30, 45, and 60 days, respectively. *p < 0.01 vs. control. †p < 0.001 vs. control.

The lung expression of p-AMPK was higher in the CS-15 group than in the CS-7 group. Notably, in the CS-30 and CS-45 groups, p-AMPK expression was higher in diaphragm muscle than in lung tissue (Figure 5). From day 7 of CS exposure onward, the lung expression of p-mTOR was lower in all CS-exposed groups than in the control group. However, that difference was most pronounced in the CS-7 and CS-45 groups. In the diaphragm muscle samples, p-mTOR expression began to increase by day 15 of CS, peaking by day 45 (Figure 5).

## Discussion

In the present study, our main objective was to characterize, at different time points, the effects induced by exposure to CS. The principal effects observed were by oxidative damage in diaphragm muscle and morphological changes in lung tissue.

The amount of neutrophils, which is associated with oxidative damage in lung tissue, was greatest on day 45 of exposure to CS. The numbers of macrophages and neutrophils are high in patients with COPD, having a direct relationship with disease severity.^(^ 21 ^)^ Our data demonstrate increases in leukocytes, including macrophages and neutrophils, from day 7 to day 45 of CS exposure, which might be related to increased cell numbers and cell proliferation, resulting in immune response activation.^(^ 22 ^)^ As observed, we confirmed that CS-induced pulmonary alterations appear to be the consequence of a primary inflammatory lesion characterized by the accumulation of alveolar macrophages and neutrophils in the lower respiratory tract as an immune response, which is crucial in inflammatory disease.^(^ 23 ^)^ It is known that ROS play an important role in the inflammatory response to CS. Oxidative stress is characterized by higher production of ROS and decreased antioxidant levels with lipid peroxidation, thiol alterations and protein carbonylation in plasma.^(^ 24 ^)^

Pulmonary emphysema is associated with intense responses in oxidative stress, which result in a direct relationship between systemic defense activity and oxidative damage.^(^ 25 ^,^ 26 ^)^ The oxidative damage and inflammation in lung tissue after exposure to CS have been widely studied. In addition, according to MacNee,^(^ 27 ^)^ oxidative stress, as quantified by measuring plasma levels of TBARS, is associated with airflow limitation. The airflow alterations play a role in the function of respiratory muscles like the diaphragm. However, our findings demonstrate that there is an increased intensity of the inflammatory response in lung tissue starting after day 45 of exposure to CS.

According to Park et al.,^(^ 10 ^)^ exposure to CS for 30 days causes significant oxidation and depletion of the glutathione pool in the lung. Those authors also concluded that the lung is a primary target of oxidative damage by cigarette smoking in the early stages, and that CS eventually exerts its oxidative effects on all organs. In our study, it was observed that CS-induced oxidative damage caused changes not only in the lungs but also in the diaphragm. We found that exposure to CS for 30-45 days was sufficient to generate higher levels of oxidative damage in skeletal muscle (the diaphragm).

A recent study showed that the main limitation found in COPD patients might be related to the mechanism of slow cardiac output associated with airflow limitation.^(^ 28 ^)^ Chiappa et al.^(^ 29 ^)^ tested conditions that improve oxygen delivery and uptake as strategies in COPD patients. The authors demonstrated that one such strategy-the use of heliox (a mixture of 79% helium and 21% oxygen)-is able to ameliorate expiratory flow limitation and dynamic hyperinflation, accelerating the dynamics of peripheral muscle utilization of oxygen as a consequence of improved delivery during high-intensity exercise in patients with moderate to severe COPD. We believed that these interactions might be linked with redox balance and inflammatory responses. One recent study suggested that, in the clinical management of acute lung injury, the use of heliox has the combined therapeutic benefits of reducing mechanical and oxidative stress, thus attenuating lung inflammation.^(^ 30 ^)^

Oxidative damage generated by exposure to CS in skeletal muscle can lead to loss of muscle function, manifesting as a loss of muscle strength and a consequent higher susceptibility to fatigue. ^(^ 1 ^,^ 31 ^)^ The present investigation is the first to provide evidence of oxidative changes induced by ROS in diaphragm muscle proteins in animals chronically exposed to CS. We found that protein oxidation was significantly increased in the diaphragm after 7 days of exposure to CS. The carbonylation of the diaphragm was highest after 30-45 days of exposure, as opposed to carbonylation in the lung, which did not peak until day 60. Our data indicate that exposure to CS primarily affects the diaphragm, which can translate to a significant loss of locomotor and respiratory muscle function in pulmonary emphysema.

According to Barreiro et al.,^(^ 1 ^)^ the effects of smoking-induced muscle protein oxidation appear at an earlier stage in the quadriceps muscle than in the respiratory muscles. These findings underscore the concept that CS per se is likely to be involved in direct tissue toxicity in the skeletal muscles of CS-exposed mice, regardless of lung and bronchial alterations. In addition, we observed that the same animals acutely exposed to CS exhibited a significant increase in TBARS, together with a reduction in muscle levels of sulfhydryl, immediately after exposure. Carbonylation is crucial to triggering activation of the oxidative pathway and promoting lipid peroxidation.

In this animal study of chronic CS exposure, we have shown that pulmonary function decreases in parallel with the duration of exposure, similar to what has been observed in humans.^(^ 32 ^)^ In addition, chronic CS exposure has been shown to cause airflow obstruction.^(^ 33 ^)^ When we analyzed the expression of p-AMPK and p-mTOR in lung tissue, we observed decreased expression of p-mTOR, a result that was expected because p-mTOR expression is associated with cell metabolism, growth, proliferation, and survival, depending on mitogenic signals, as well as on the availability of nutrients and energy.

The increased expression of p-mTOR observed in the diaphragm from day 15 to day 45 of CS exposure can be explained by the possible increase in muscle protein synthesis related to a state of physiological stress.^(^ 34 ^)^ In a rat model of CS exposure, Kozma et al.^(^ 5 ^)^ demonstrated that airway resistance and respiratory system resistance were higher in exposed animals than in unexposed animals. This increase in airway resistance might result in a greater diaphragmatic work, which would explain the increased diaphragm expression of p-mTOR in our CS-15, CS-30, and CS-45 groups, given that p-mTOR expression is known to be elevated in situations of muscle hypertrophy. ^(^ 35 ^)^ In our CS-60 group, there was a significant reduction in p-mTOR expression, which was an expected result, because myopathy is associated with reduced expression of p-mTOR.^(^ 36 ^)^ Such myopathy is common in chronic lung diseases.^(^ 1 ^)^ However, in our study, the expression of p-AMPK was increased only from day 30 to day 45 of CS exposure. This fact might be explained by the fact that the increased p-AMPK expression was accompanied by an increase in oxidative stress, which is clear when we look at the increase in carbonyl by day 30 of CS exposure. Increasing evidence suggests that p-AMPK can be activated by an increase in intracellular ROS in many cell types.^(^ 12 ^)^ Accordingly, whether the ROS-sensitive p-AMPK signaling pathway is involved in toxic smoke-induced lung inflammation remains to be investigated.

Perang et al.^(^ 37 ^)^ were the first to report a detailed AMPK signaling pathway responsible for inducing interleukin (IL)-8 expression by toxic smoke exposure in lung epithelial cells. In this pathway, increased intracellular levels of ROS level constitute the vital trigger, because removal of intracellular ROS by N-acetyl-cysteine reduced the activation of AMPK, c-Jun N-terminal kinase, and extracellular signal-regulated kinase, as well as the induction of IL-8.^(^ 37 ^)^ Previous studies have reported that toxic smoke can increase the intracellular ROS level in lung cells, although the mechanism remains unclear.^(^ 38 ^)^

In conclusion, our study shows, for the first time, that oxidative alterations in muscle proteins occur in the diaphragm as early as day 7 days of exposure to CS. In addition, this event occurred concomitantly with the parenchymal abnormalities induced by CS in the lungs, suggesting a direct toxic effect of CS on skeletal muscle proteins. However, our data also make it more obvious that pulmonary emphysema is a complex disease that has a negative impact on the whole body. Furthermore, we found that the oxidative damage caused by CS exposure occurs first in skeletal muscle and then in lung tissue.
