# Supplementary material for: Oxidative damage induced by cigarette smoke exposure in mice: impact on lung tissue and diaphragm muscle
Source: J Bras Pneumol. 2014 Jul-Aug;40(4):411–20. doi: 10.1590/S1806-37132014000400009 (PMC4201172; doi:10.1590/S1806-37132014000400009)
Supplement: Translation

# Translation: Dano oxidativo induzido por exposição a fumaça de cigarro em camundongos: impacto sobre o pulmão e o músculo diafragma*

**Authors:** Samanta Portão de Carlos, Alexandre Simões Dias, Luiz Alberto Forgiarini, Patrícia Damiani Patricio, Thaise Graciano, Renata Tiscoski Nesi, Samuel Valença, Adriana Meira Guntzel Chiappa, Gerson Cipriano, Claudio Teodoro de Souza, Gaspar Rogério da Silva Chiappa

**Affiliations:** Universidade do Sul de Santa Catarina, Criciúma (SC) Brasil; Programa de Pós-Graduação em Ciências do Movimento e Ciências Pneumológicas, Universidade Federal do Rio Grande do Sul, Porto Alegre (RS) Brasil; Universidade Metodista, Instituto Porto Alegre – IPS – Porto Alegre (RS) Brasil; Universidade do Sul de Santa Catarina, Criciúma (SC) Brasil; Universidade do Sul de Santa Catarina, Criciúma (SC) Brasil; Instituto de Ciências Biomédicas, Universidade Federal do Rio de Janeiro, Rio de Janeiro (RJ) Brasil; Laboratório de Pesquisa em Fisiopatologia do Exercício, Departamento de Cardiologia, Hospital de Clínicas de Porto Alegre, Porto Alegre (RS) Brasil; Unidade de Terapia Intensiva, Hospital de Clínicas de Porto Alegre, Porto Alegre (RS) Brasil; Programa de Ciências da Saúde e Tecnologias, Departamento de Fisioterapia, Universidade de Brasília, Brasília (DF) Brasil; Departamento de Fisioterapia, Universidade do Sul de Santa Catarina, Criciúma (SC) Brasil; Laboratório de Pesquisa em Fisiopatologia do Exercício, Departamento de Cardiologia, Hospital de Clínicas de Porto Alegre, Porto Alegre (RS) Brasil; e Grupo de Pesquisa em Epidemiologia e Saúde Pública, Faculdade da Serra Gaúcha, Caxias do Sul (RS) Brasil

**Keywords:** Estresse oxidativo, Camundongos, Sistema respiratório, Poluição por fumaça de tabaco, Inflamação

## Abstract

**OBJETIVO:**

Avaliar o dano oxidativo (oxidação lipídica, oxidação proteica, *thiobarbituric acid-reactive substances *[TBARS, substâncias reativas ao ácido tiobarbitúrico], e carbonilação) e inflamação (expressão de *phosphorylated AMP-activated protein kinase* e de *phosphorylated mammalian target of rapamycin* (p-AMPK e p-mTOR, respectivamente) em tecido pulmonar e músculos do diafragma em camundongos C57BL/6 machos expostos à fumaça de cigarro (FC) por 7, 15, 30, 45 ou 60 dias.

**MÉTODOS:**

Trinta e seis camundongos machos da espécie C57BL/6 foram divididos em seis grupos (n = 6/grupo): grupo controle e 5 grupos expostos a FC por 7, 15, 30, 45 e 60 dias, respectivamente.

**RESULTADOS:**

Comparados aos camundongos controle, os camundongos expostos à FC apresentaram menor peso corporal em 30 dias. Nos camundongos expostos à FC (comparados aos controle) as maiores diferenças (aumentos) nos níveis de TBARS foram observados no dia 7 no músculo diafragma, comparado ao dia 45 em tecido pulmonar; as maiores diferenças (aumentos) nos níveis de carbonilas foram observados no dia 7 em ambos os tipos de tecido; e os níveis de sulfidrilas foram menores, nos dois tipos de tecidos, em todos os tempos. No tecido pulmonar e no músculo diafragma, a expressão de p-AMPK exibiu um comportamento semelhante ao dos níveis de TBARS. A expressão de p-mTOR foi maior que o valor controle nos dias 7 e 15 no tecido pulmonar, assim como no dia 45 no músculo diafragma.

**CONCLUSÕES:**

Nossos dados demonstram que a exposição à FC produz dano oxidativo tanto no tecido pulmonar quanto (primariamente) no tecido muscular, tendo um efeito adicional no músculo respiratório, como é frequentemente observado em fumantes com DPOC.

## Introdução

A fumaça de cigarro (FC) contém um grande número de oxidantes que têm efeitos adversos sobre os tecidos por meio de dano oxidativo.^(^ 1 ^,^ 2 ^)^ Sabe-se que a FC ativa as células inflamatórias, o que pode também aumentar a produção de oxidantes por células polimorfonucleares nos tecidos, desencadeando o estresse oxidativo, etapa crucial na patogênese do dano tecidual induzido pela FC.^(^ 3 ^-^ 6 ^)^ Os efeitos combinados de aumento da degradação proteolítica, aumento da morte celular e redução do remodelamento pulmonar levam a alterações enfisematosas nos pulmões.^(^ 7 ^)^ Estudos mostraram que, no sangue de fumantes,^(^ 8 ^,^ 9 ^)^ bem como em vários órgãos de animais cronicamente expostos à FC,^(^ 10 ^)^ há aumentos da peroxidação lipídica, da carbonilação de proteínas, da oxidação de tióis e da oxidação do DNA.

Há evidências de que dois fatores centrais estão envolvidos na lesão direta ou inflamação sistêmica induzida pela FC: a *phosphorylated AMP-activated protein kinase* e o *phosphorylated mammalian target of rapamycin* (p-AMPK e p-mTOR, respectivamente). Um estudo recente mostrou que a ativação da p-AMPK inibe ou promove a inflamação, dependendo do estímulo. ^(^ 11 ^)^ Há também evidências crescentes de que, em muitos tipos de células, um aumento dos níveis intracelulares de espécies reativas de oxigênio (ERO) pode ativar a p-AMPK.^(^ 12 ^)^ O mTOR, que é um importante integrador de estímulos ambientais, controla o metabolismo, crescimento, proliferação e sobrevivência celulares, dependendo de sinais mitogênicos, bem como da disponibilidade de nutrientes e de energia. Recentemente ficou claro que a sinalização do mTOR desempenha um papel central na regulação de aspectos básicos do comportamento das células e do organismo, e sua desregulação associa-se fortemente à progressão de numerosas doenças humanas proliferativas e metabólicas, incluindo o câncer, a obesidade, o diabetes tipo 2 e a síndrome do hamartoma.^(^ 13 ^)^

É muito importante elucidar o possível dano oxidativo induzido pela FC diretamente no músculo esquelético, bem como as anormalidades estruturais relacionadas e a relação direta entre a p-AMPK e o p-mTOR, dois fatores associados à inflamação. Portanto, o objetivo deste estudo em animais foi avaliar o dano oxidativo e a inflamação no parênquima pulmonar e no músculo diafragma após 7, 15, 30, 45 e 60 dias de exposição à FC.

## Métodos

Neste estudo, utilizamos 36 camundongos machos da espécie C57BL/6 com idade de dois meses e peso de 30-35 g. Os animais foram utilizados e cuidados de acordo com a Diretiva 86/609/CEE, de 24 de novembro de 1986, do Conselho das Comunidades Europeias. Os procedimentos adotados neste estudo foram aprovados pelo Comitê de Ética em Pesquisa da Universidade do Extremo Sul de Santa Catarina, localizada em Criciúma. Os camundongos foram mantidos em ambiente com temperatura e umidade controladas (umidade de 70%; 20 ± 2°C), em ciclo claro-escuro de 12 h, e tinham livre acesso a água e ração (Nuvilab CR1; Nuvital Nutrientes Ltda., Colombo, Brasil). Os animais foram verificados periodicamente para confirmar a ausência de patógenos. Para os ensaios bioquímicos, os camundongos foram divididos aleatoriamente em 6 grupos (n = 6/grupo): grupo controle e 5 grupos expostos a FC por 7, 15, 30, 45 e 60 dias (designados FC-7, FC-15, FC-30, FC-45 e FC-60, respectivamente).

Foram utilizados cigarros comerciais com filtro (Marlboro^TM^ Red, 8 mg de alcatrão e 0,6 mg de nicotina por cigarro; Philip Morris Products, Richmond, VA, EUA).^(^ 14 ^,^ 15 ^)^ Os animais do estudo foram expostos à fumaça emitida pela queima de 12 cigarros ao dia por 7, 15, 30, 45 e 60 dias, conforme descrito anteriormente por Menegali et al.^(^ 3 ^)^ Em suma, os animais foram colocados em uma câmara de inalação coberta (40 cm de comprimento, 30 cm de largura e 25 cm de altura), posicionada sob um exaustor. Um cigarro foi acoplado a uma seringa plástica de 60 mL de modo que cada baforada pudesse ser colhida e em seguida expelida para a câmara de exposição. Um litro de fumaça (20 baforadas de 50 mL) foi aspirado de cada cigarro, sendo que cada baforada foi imediatamente injetada na câmara de inalação. Os animais foram mantidos nessa condição de fumaça-ar (3% de fumaça) por 6 min. Retirou-se então a tampa da câmara de inalação e ligou-se o exaustor, que evacuou a fumaça em 60 s. Esse processo foi imediatamente repetido. Um total de quatro cigarros foi assim "fumado" em cada tratamento. Os camundongos foram submetidos a esses tratamentos com 4 cigarros três vezes ao dia (manhã, meio-dia e tarde), resultando na exposição à FC por 72 min (12 cigarros por dia). ^(^ 16 ^)^ Cada cigarro fumado produziu 300 mg/m^3^ de particulados totais na câmara de exposição.^(^ 3 ^)^ Os animais foram sacrificados por deslocamento cervical 24 h após a última exposição à FC. As amostras de tecido pulmonar e do músculo diafragma foram homogeneizadas em tampão. Os homogeneizados foram centrifugados a 1000 × *g* por 10 min a 4°C, e os sobrenadantes foram armazenados a −70°C para posterior utilização nos experimentos.

Para a análise histológica, selecionaram-se todos os animais de cada grupo. O ventrículo direito foi submetido a perfusão com solução salina estéril (0,9%) a fim de remover o sangue do pulmão. O pulmão direito foi fixado (por infusão suave de formalina tamponada com fosfato a 4% [pH 7,2], a 25 cmH_2_O, por 2 min, através de cateter traqueal), após o que o mesmo foi retirado e pesado. Os pulmões inflados foram fixados por 48 h e então incluídos em parafina. Cortes seriados sagitais (5 µm) foram obtidos para as análises histológicas e morfométricas. Realizou-se a quantificação de macrófagos e neutrófilos nos alvéolos. Para cada grupo, foram analisados 30 campos microscópicos (10 campos aleatórios, de 26.000 mm^2^ cada, em 3 diferentes cortes do pulmão direito). A contagem do número de macrófagos e neutrófilos (células/mm^2)^ foi realizada em um microscópio de fluorescência (BH-2; Olympus, Tóquio, Japão) equipado com objetiva de 40×.^(^ 3 ^)^

O dano oxidativo foi avaliado pela quantificação de sulfidrilas, carbonilas proteicas e malondialdeído. O conteúdo total de tióis foi determinado pelo método do ácido 5,5'-ditiobis-(2-nitrobenzoico) - reagente DTNB (Sigma, St. Louis, MO, EUA). O teste do reagente DTNB foi realizado conforme condições descritas anteriormente.^(^ 17 ^)^ Em suma, 30 µL de uma amostra foram misturados a 1 mL de PBS e 1 mM de EDTA (pH 7,5). A reação foi iniciada pela adição de 30 µL de solução estoque de DTNB (10 mM em PBS). As amostras controle, que não incluíam DTNB ou proteína, foram processadas simultaneamente. Após 30 min de incubação em temperatura ambiente, leu-se a absorbância a 412 nm e mediram-se as quantidades de ácido 5-tio-2-nitrobenzoico (TNB) formado (equivalentes à quantidade de grupos sulfidrilas). As carbonilas proteicas foram determinadas pelo método espectrofotométrico utilizando 2,4-dinitrofenil-hidrazina (DNPH), conforme descrito por Levine et al.^(^ 18 ^)^ Em suma, as amostras contendo ou ácido clorídrico 2 N ou DNPH foram passadas através de colunas contendo Sephadex G-10 e lavadas com ácido clorídrico 2 N. O efluente foi coletado e misturado a cloridrato de guanidina, após o que se determinou a absorbância a 360 nm em espectrofotômetro (SP 1105; Shanghai Spectrum Instruments Co., Ltd., Xangai, China). A diferença na absorbância com e sem DNPH foi calculada para todas as amostras. Os valores foram expressos em unidades molares utilizando-se o coeficiente de extinção de 22.000 [M-1]. A determinação do malondialdeído, um importante indicador de peroxidação lipídica, foi realizada por espectrofotometria do produto de cor rosa resultante das *thiobarbituric acid-reactive substances* (TBARS, substâncias reativas ao ácido tiobarbitúrico). As TBARS totais, como *proxy* para peroxidação lipídica (níveis de malondialdeído), foram expressas em mmol/mg de proteína.^(^ 19 ^)^

Para Western blotting, os homogeneizados de pulmão foram preparados a partir de pulmões congelados, utilizando-se um tampão de lise de tecido (50 mM de TRIS, pH 8,0; 5 mM de EDTA; 150 mM de NaCl; 1% de detergente não iônico; 0,5% de desoxicolato de sódio; e 0,1% de dodecilsulfato de sódio) e um coquetel de inibidores de protease (Sigma). Os lisados foram clarificados por centrifugação a 13.000 g por 15 min a 4°C; 10-30 g de proteína foram separados por SDS-PAGE em gel a 10% ou 15%; e a expressão de p-AMPK e p-mTOR foi analisada por immunoblot. A detecção da imunorreatividade foi realizada por quimiluminescência (ECL; Amersham Biosciences, Buckinghamshire, Reino Unido). A densidade das bandas foi determinada utilizando-se um densitômetro de imagem e analisada com o software do próprio aparelho ((GS-700 e Quantity One; Bio-Rad Laboratories, Hercules, CA, EUA).^(^ 20 ^)^

Os dados foram expressos em média ± erro padrão da média. Para comparar as médias entre os grupos, utilizou-se ANOVA de um fator seguida do teste *post hoc* HSD de Tukey para comparações múltiplas. O nível de significância adotado foi de p < 0,05. O software utilizado para a análise dos dados foi o *Statistical Package for the Social Sciences*, versão 18.0 para Windows (SPSS Inc., Chicago, IL, EUA). O tamanho da amostra foi baseado em estudos anteriores realizados em nosso laboratório,^(^ 3 ^)^ em que abordagens semelhantes foram empregadas.

## Resultados

Entre os camundongos avaliados no presente estudo, a taxa de sobrevivência foi de 100%. Em comparação aos valores basais, o peso corporal dos animais diminuiu após 30, 45 e 60 dias de exposição à FC (27 ± 1 vs. 23 ± 0,8 g; p < 0,01, 26 ± 0,5 vs. 22 ± 0,4 g; p < 0,01, e 25 ± 0,7 vs. 20 ± 0,3 g; p < 0,001, respectivamente). Além disso, o peso corporal dos camundongos do grupo FC-60 foi significativamente menor que o dos camundongos controle, sendo também significativamente menor que o dos camundongos dos grupos FC-30 e FC-45 (p < 0,001 para todos).

Na análise histológica, as amostras de tecido pulmonar dos camundongos controle apresentaram septos alveolares finos e alvéolos normais, enquanto as dos camundongos que foram expostos à FC apresentaram destruição dos septos alveolares (iniciando-se no 15º dia de exposição), alargamento dos alvéolos e presença de macrófagos alveolares (Figura 1A). O alargamento dos alvéolos foi significativamente maior no grupo FC-45 (Figura 1A). Conforme mostrado na Figura 1B, os números tanto de macrófagos quanto de neutrófilos nos grupos FC aumentaram significativamente (em comparação aos observados para o grupo controle) em 7 dias de exposição à FC (p < 0,01). Porém, a diferença no número de neutrófilos foi mais pronunciada após 45 dias de exposição (p < 0,001).

**Figura 1:** Em A, fotomicrografias de amostras de tecido pulmonar de camundongos expostos a fumaça de cigarro mostrando aumento dos espaços aéreos (EA) resultante da consolidação alveolar durante o desenvolvimento de enfisema pulmonar (aumento, 40×): a, grupo controle; b, grupo exposto por 7 dias; c, grupo exposto por 15 dias; d, grupo exposto por 30 dias; e, grupo exposto por 45 dias; e f, grupo exposto por 60 dias. Em B, média ± EPM do número de macrófagos e de neutrófilos (células/mm2). *p < 0,001 vs. controle para macrófagos. †p < 0,001 vs. controle para neutrófilos. ‡p < 0,001 vs. basal para neutrófilos.

As Figuras 2, 3 e 4, respectivamente, mostram peroxidação lipídica, carbonilas proteicas e conteúdo de sulfidrila nas amostras de tecido pulmonar e nas amostras do músculo diafragma. Em ambos os tipos de tecido, as TBARS totais aumentaram após 7 dias de exposição à FC, da mesma foram que os níveis de carbonilas. Nos grupos FC-7, FC-15 e FC-45, houve diferenças entre as amostras de tecido pulmonar e as amostras do músculo diafragma em termos do grau de aumento dos níveis de carbonilas. No grupo FC-15, os níveis de TNB foram significativamente menores no tecido pulmonar que no músculo diafragma. Porém, em 7 dias de exposição à FC, os níveis de TNB foram menores que os valores controle em ambos os tipos de tecido.

**Figura 2:** Média ± EPM dos níveis de thiobarbituric acid-reactive substances (TBARS) no tecido pulmonar e no músculo diafragma em 6 grupos de camundongos: grupo controle e 5 grupos expostos a fumaça de cigarro por 7, 15, 30, 45 e 60 dias, respectivamente. *p < 0,05 vs. controle no tecido pulmonar. † p < 0,05 vs. controle no músculo diafragma. ‡ p < 0,05 vs. tecido pulmonar.

**Figura 3:** Média ± EPM dos níveis de carbonilas no tecido pulmonar e no músculo diafragma em 6 grupos de camundongos: grupo controle e 5 grupos expostos a fumaça de cigarro por 7, 15, 30, 45 e 60 dias, respectivamente. p < 0,05 vs. controle no tecido pulmonar. † p < 0,05 vs. controle no músculo diafragma. ‡ p < 0,05 vs. tecido pulmonar.

**Figura 4:** Média ± EPM dos níveis de ácido 5-tio-2- nitrobenzoico (TNB) no tecido pulmonar e no músculo diafragma em 6 grupos de camundongos: grupo controle e 5 grupos expostos a fumaça de cigarro por 7, 15, 30, 45 e 60 dias, respectivamente. * p < 0,05 vs. controle no tecido pulmonar. † p < 0,05 vs. controle no músculo diafragma. ‡ p < 0,05 vs. tecido pulmonar.

A expressão de p-AMPK no pulmão foi maior no grupo FC-15 que no grupo FC-7. Notadamente, nos grupos FC-30 e FC-45, a expressão de p-AMPK foi maior no músculo diafragma que no tecido pulmonar (Figura 5). Do 7º dia de exposição à FC em diante, a expressão de p-mTOR no pulmão foi menor em todos os grupos expostos à FC que no grupo controle. Porém, essa diferença foi mais pronunciada nos grupos FC-7 e FC-45. Nas amostras do músculo diafragma, a expressão de p-mTOR começou a aumentar em 15 dias de exposição à FC, atingindo seu pico em 45 dias (Figura 5).

**Figura 5:** Em A e B, média ± EPM para expressão de phosphorylated AMP-activated protein kinase (p-AMPK) no tecido pulmonar e no músculo diafragma, respectivamente. Em C e D, média ± EPM para expressão de phosphorylated mammalian target of rapamycin (p-mTOR) no tecido pulmonar e no músculo diafragma, respectivamente. Os dados são referentes a 6 grupos de camundongos: grupo controle e 5 grupos expostos a fumaça de cigarro por 7, 15, 30, 45 e 60 dias, respectivamente. *p < 0,01 vs. controle. †p < 0,001 vs. controle.

## Discussão

No presente estudo, nosso principal objetivo foi caracterizar, em diferentes momentos, os efeitos induzidos pela exposição à FC. Os principais efeitos observados foram dano oxidativo no músculo diafragma e alterações morfológicas no tecido pulmonar.

A quantidade de neutrófilos, que está associada ao dano oxidativo no tecido pulmonar, alcançou seu maior valor em 45 dias de exposição à FC. Os números de macrófagos e de neutrófilos são elevados em pacientes com DPOC, apresentando relação direta com a gravidade da doença.^(^ 21 ^)^ Nossos dados demonstram aumentos de leucócitos, incluindo macrófagos e neutrófilos, do 7º ao 45º dia de exposição à FC, o que pode estar relacionado a aumento do número de células e proliferação celular, resultando na ativação da resposta imune.^(^ 22 ^)^ Conforme observado, confirmamos que as alterações pulmonares induzidas pela FC parecem ser consequência de uma lesão inflamatória primária caracterizada pelo acúmulo de macrófagos alveolares e neutrófilos no trato respiratório inferior como resposta imune, que é crucial na doença inflamatória.^(^ 23 ^)^ Sabe-se que as ERO desempenham um papel importante na resposta inflamatória à FC. O estresse oxidativo é caracterizado pela maior produção de ERO e diminuição dos níveis de antioxidantes, com a peroxidação lipídica, alterações tióis e carbonilação de proteínas no plasma.^(^ 24 ^)^

O enfisema pulmonar está associado a respostas intensas ao estresse oxidativo, que resultam em uma relação direta entre a atividade de defesa sistêmica e o dano oxidativo.^(^ 25 ^,^ 26 ^)^ O dano oxidativo e a inflamação no tecido pulmonar após exposição à FC têm sido amplamente estudados. Além disso, de acordo com MacNee,^(^ 27 ^)^ o estresse oxidativo, quantificado por meio da determinação dos níveis plasmáticos de TBARS, está associado à limitação do fluxo aéreo. As alterações do fluxo aéreo desempenham um papel na função dos músculos respiratórios como o diafragma. Porém, nossos achados demonstram que há aumento da intensidade da resposta inflamatória no tecido pulmonar começando após 45 dias de exposição à FC.

De acordo com Park et al.,^(^ 10 ^)^ a exposição à FC por 30 dias causa oxidação significativa e depleção do *pool *de glutationa no pulmão. Esses autores também concluíram que o pulmão é um alvo primário do dano oxidativo pelo tabagismo nos estágios iniciais e que, em algum momento, a FC exerce seus efeitos oxidativos em todos os órgãos. Em nosso estudo, observou-se que o dano oxidativo induzido pela FC causou alterações tanto nos pulmões quanto no diafragma. Verificou-se que a exposição à FC por 30-45 dias foi suficiente para gerar maiores níveis de dano oxidativo em músculo esquelético (diafragma).

Um estudo recente mostrou que a principal limitação encontrada em pacientes com DPOC pode estar relacionada ao mecanismo de débito cardíaco lento associado à limitação do fluxo aéreo.^(^ 28 ^)^ Chiappa et al.^(^ 29 ^)^ testaram condições que melhoram a oferta e o consumo de oxigênio como estratégias em pacientes com DPOC. Os autores demonstraram que uma dessas estratégias - o uso de heliox (uma mistura de 79% de hélio e 21% de oxigênio) - é capaz de melhorar a limitação do fluxo expiratório e a hiperinsuflação dinâmica, acelerando a dinâmica de utilização de oxigênio pela musculatura periférica em consequência do aumento da oferta durante exercícios de alta intensidade em pacientes com DPOC moderada a grave. Acreditamos que essas interações podem estar ligadas ao equilíbrio redox e às respostas inflamatórias. Um estudo recente sugeriu que, no manejo clínico da lesão pulmonar aguda, o uso de heliox apresenta benefícios terapêuticos combinados de redução do estresse mecânico e oxidativo, atenuando assim a inflamação pulmonar.^(^ 30 ^)^

O dano oxidativo gerado pela exposição à FC no músculo esquelético pode levar à perda da função muscular, manifestando-se como perda de força muscular e consequente maior susceptibilidade à fadiga.^(^ 1 ^,^ 31 ^)^ A presente investigação é a primeira a fornecer evidências de alterações oxidativas induzidas pelas ERO nas proteínas do músculo diafragma em animais cronicamente expostos à FC. Verificou-se que a oxidação proteica estava significativamente aumentada no diafragma após 7 dias de exposição à FC. Os maiores níveis de carbonilação do diafragma foram observados após 30-45 dias de exposição, em oposição aos níveis de carbonilação no pulmão, que só atingiram seu pico em 60 dias. Nossos dados indicam que a exposição à FC afeta principalmente o diafragma, o que pode se traduzir em perda significativa da função locomotora e da musculatura respiratória em enfisema pulmonar.

De acordo com Barreiro et al.,^(^ 1 ^)^ os efeitos da oxidação proteica muscular induzida pelo tabaco no músculo quadríceps surgem em uma fase anterior, em comparação aos observados nos músculos respiratórios. Esses achados reforçam o conceito de que a FC, por si só, provavelmente está envolvida na toxicidade tecidual direta da musculatura esquelética dos camundongos expostos à FC, independentemente das alterações pulmonares e brônquicas. Além disso, observou-se que os mesmos animais agudamente expostos à FC apresentaram aumento significativo das TBARS, juntamente com redução dos níveis musculares de sulfidrila, imediatamente após a exposição. A carbonilação é fundamental para desencadear a ativação da via oxidativa e promover a peroxidação lipídica.

Neste estudo sobre exposição crônica à FC em animais, mostramos que a função pulmonar diminui com o tempo de exposição, à semelhança do que foi observado em humanos.^(^ 32 ^)^ Além disso, há relatos de que a exposição crônica à FC causa obstrução ao fluxo aéreo.^(^ 33 ^)^ Quando analisamos a expressão de p-AMPK e p-mTOR no tecido pulmonar, observamos diminuição da expressão de p-mTOR, resultado que era esperado, pois a expressão de p-mTOR está associada ao metabolismo, crescimento, proliferação e sobrevivência celulares, dependendo de sinais mitogênicos, bem como da disponibilidade de nutrientes e de energia.

O aumento da expressão de p-mTOR observado no diafragma do 15º ao 45º dia de exposição à FC pode ser explicado pelo possível aumento da síntese proteica muscular relacionado a um estado de estresse fisiológico.^(^ 34 ^)^ Em um modelo de exposição à FC em ratos, Kozma et al.^(^ 5 ^)^ demonstraram que a resistência das vias aéreas e a resistência do sistema respiratório eram maiores nos animais expostos que nos animais não expostos. Esse aumento da resistência das vias aéreas pode resultar em maior trabalho diafragmático, o que explicaria o aumento da expressão de p-mTOR no diafragma em nossos grupos FC-15, FC-30 e FC-45, uma vez que se sabe que a expressão de p-mTOR é elevada em situações de hipertrofia muscular.^(^ 35 ^)^ Em nosso grupo FC-60, houve redução significativa da expressão de p-mTOR, o que foi um resultado esperado, pois a miopatia está associada à redução da expressão de p-mTOR.^(^ 36 ^)^ Essa miopatia é comum em doenças pulmonares crônicas.^(^ 1 ^)^ Porém, em nosso estudo, a expressão de p-AMPK encontrava-se aumentada apenas do 30º ao 45º dia de exposição à FC. Esse fato pode ser explicado pelo fato de que o aumento da expressão de p-AMPK foi acompanhado por aumento do estresse oxidativo, que fica claro quando olhamos para o aumento das carbonilas em 30 dias de exposição à FC. Evidências crescentes sugerem que a p-AMPK pode ser ativada por aumento de ERO intracelulares em muitos tipos de células.^(^ 12 ^)^ Assim, o envolvimento ou não da via de sinalização da p-AMPK sensível a ERO na inflamação pulmonar induzida pela fumaça tóxica ainda requer investigação.

Perang et al.^(^ 37 ^)^ foram os primeiros a relatar uma detalhada via de sinalização da AMPK responsável pela indução da expressão da interleucina (IL)-8 por exposição à fumaça tóxica em células epiteliais pulmonares. Nessa via, o aumento dos níveis intracelulares de ERO constitui o gatilho vital, pois a remoção de ERO intracelulares pela N-acetil-cisteína reduziu a ativação da AMPK, da c-Jun N-terminal quinase e da quinase regulada por sinal extracelular, bem como a indução de IL-8. ^(^ 37 ^)^ Estudos anteriores relataram que a fumaça tóxica pode aumentar o nível intracelular de ERO em células pulmonares, embora o mecanismo permaneça obscuro.^(^ 38 ^)^

Em conclusão, nosso estudo mostra, pela primeira vez, que as alterações oxidativas nas proteínas musculares ocorrem no diafragma já em 7 dias de exposição à FC. Além disso, esse evento ocorreu concomitantemente às anormalidades parenquimatosas induzidas pela FC nos pulmões, sugerindo um efeito tóxico direto da FC nas proteínas do músculo esquelético. Porém, nossos dados também tornam mais óbvio que o enfisema pulmonar é uma doença complexa que tem impacto negativo no corpo inteiro. Ademais, verificou-se que o dano oxidativo causado pela exposição à FC ocorre primeiro no músculo esquelético e então no tecido pulmonar.
